# Shaping the Archaeal Cell Envelope

**DOI:** 10.1155/2010/608243

**Published:** 2010-07-07

**Authors:** Albert F. Ellen, Behnam Zolghadr, Arnold M. J. Driessen, Sonja-Verena Albers

**Affiliations:** ^1^Centre for Integrative Biology, Microbial Genomics, Via delle Regole 101, 38123 Mattarello, Italy; ^2^Department of Molecular Microbiology, Groningen Biomolecular Sciences and Biotechnology Institute, The Zernike Institute for Advanced Materials, University of Groningen, Kerklaan 30, 9751 NN Haren, The Netherlands; ^3^Molecular Biology of Archaea, Max Planck Institute for terrestrial Microbiology, Karl-von-Frisch-Straße 10, 35043 Marburg, Germany

## Abstract

Although archaea have a similar cellular organization as other prokaryotes, the lipid composition of their membranes and their cell surface is unique. Here we discuss recent developments in our understanding of the archaeal protein secretion mechanisms, the assembly of macromolecular cell surface structures, and the release of S-layer-coated vesicles from the archaeal membrane.

## 1. The Archaeal Cell Envelope

The ability of many archaea to endure extreme conditions in hostile environments intrigues researchers to study the molecular mechanisms and specific adaptations involved. Very early, it was realized that the structure of the archaeal cell envelope differs substantially from that of bacteria [1]. With the only exception of *Ignicoccus* which exhibits an outer membrane enclosing a huge periplasmic space [2], known archaea possess only a single membrane. This cytoplasmic membrane is enclosed by an S-layer, a two-dimensional protein crystal that fully covers the cells (see review Jarrell et al. in this issue). In contrast to bacterial ester lipids, archaeal lipids consist of repeating isoprenyl groups linked to a glycerol backbone through an ether linkage [3, 4]. These lipids typically form diether bilayer membranes similar to membranes of eukarya and bacteria. Hyperthermo-acidophiles contain tetraether lipids that consist of C_40_ isoprenoid acyl chains that span the membrane entirely forming a monolayer membrane [5]. These membranes are extremely proton impermeable and enable these organisms to survive under conditions that the extracellular pH is up to 4 units below that of the cytoplasm [6]. Another peculiarity is that most of the extracellular proteins of archaea are glycosylated via N- and O-glycosylation. Finally, Archaea do not produce any murein, and only some methanogenic species are known to produce pseudomurein [7]. 

As the archaeal cell surface is so different from that of bacteria and eukarya, unique mechanisms must exist to form and shape it. Until recently most of our knowledge of protein secretion and on the assembly of the cell surface components in archaea was obtained by comparative genomic studies. However, in recent years tremendous progress has been made in our understanding of the assembly and function of cell surface structures and both the structural and functional basis of protein translocation across the archaeal membrane. Here we will discuss these topics with an emphasis on the cell surface structures.

## 2. Protein Secretion

### 2.1. Transport of Unfolded Proteins Across the Cytoplasmic Membrane

**Figure 1:**
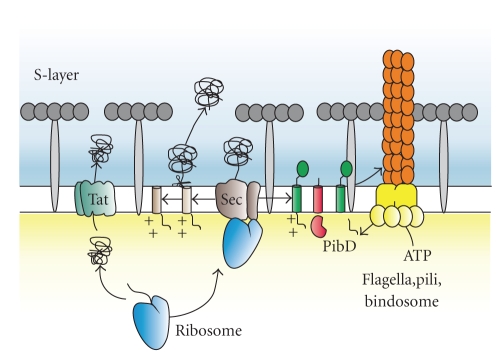
Model of the archaeal cell envelope showing different characterized secretion pathways. Proteins synthesized at the ribosome can follow several routes to the exterior of the cell. During co-translational translocation, the ribosome-nascent chain complex is targeted to the SecYE*β* complex by the signal recognition particle. At the SecYE*β* complex protein synthesis and translocation across the cytoplasm membrane occurs simultaneously. In the case of a preprotein with a class I signal peptide, the signal peptide is removed during translocation and the protein is released and folds at the external face of the membrane. Class III signal peptide containing proteins translocated via the SecYE*β* complex are processed by PibD and subsequently assembled into a flagellum, pilus, bindosome or so far unknown cell surface structures. Alternatively, folded proteins are transported across the cytoplasmic membrane via the Twin arginine translocase pathway.

The ability to transport proteins across membranes is vital for cell viability. In general, the systems found in archaea that mediate protein transport across the cytoplasmic membrane are similar to those of bacteria. In archaea most proteins are secreted across the cytoplasmic membrane by the general secretion (Sec) or Twin arginine translocase (Tat) route (see Figure 1). The Sec pathway consists of a universally conserved translocation complex embedded in the membrane, which is termed SecYEG in bacteria and Sec61p in the endoplasmic reticulum (ER) of eukaryotes. The Sec system handles the transport of unfolded proteins but is also required for the integration of membrane proteins into the cytoplasmic membrane [8]. In bacteria, the SecYEG complex either associates with the ribosome for cotranslational membrane protein insertion or with the motor protein SecA, to catalyze posttranslational protein translocation. In the ER, Sec61p associates with the ribosome for co-translational protein translocation and membrane protein insertion and Sec61p associates with the Sec63p complex and the ER luminal chaperone BiP for post-translational protein translocation. The core of the protein-conducting channel is composed of two essential components, SecY and SecE in bacteria and Sec61*α* and Sec61*γ* in eukaryotes [9]. Both proteins are found in all archaea but the third, nonessential component, that is, SecG in bacteria or Sec61*β* in eukaryotes, was identified only after extensive bioinformatic analyses [10, 11]. In this respect, the archaeal SecG homolog is more related to the eukaryotic Sec61*β* than to the bacterial SecG. Therefore, the archaeal translocon is often referred to as the SecYE*β* complex [12]. The exact composition of the minimal protein translocase of Archaea has, however, remained unclear. Archaea lack a homolog of the bacterial SecA motor protein, a protein that is well conserved among bacteria and the chloroplast thylakoid [8]. Likewise, Archaea also do not contain homologs of the eukaryal Sec63p complex, but they do contain DnaK (or Hsp70) chaperones homologous to BiP. These chaperones fulfill general functions in protein folding but in analogy with the ER, a BiP homolog involved in protein translocation would need to be extracellular. However, no archaeal Hsp70 homolog has been detected extracellularly and of course the energy source ATP would be absent. Therefore, it is generally assumed that protein translocation is co-translationally coupled to chain elongation at the ribosome [13]. However, in the euryarchaeon *Haloferax volcanii,* it was noted that some proteins are present as fully synthesized signal peptide bearing precursors in the cytoplasm before they are secreted. Based on this finding, it has been proposed that post-translational protein secretion also exists in archaea [14]. Interestingly, euyarchaeota contain a homolog of the bacterial SecDF protein complex [15], whereas this protein is absent from crenarchaeota. The exact role of SecDF is unknown, but it has been implicated in the proton motive force-dependent release of translocated proteins from the periplasmic face of the membrane. SecDF is not essential for translocation per se, but it enhances the rate of translocation. Other suggested roles of SecDF are that it may act on the SecA ATPase catalytic cycle but since SecA is absent from archaea such a role seems unlikely.

The structural analysis of the *Methanocaldococcus jannashii* SecYE*β* heterotrimer [12] has provided important insights in how this channel may function in protein translocation. The main subunit SecY consists of two halves with an internal pseudo-twofold symmetry. These two halves comprise transmembrane segments (TMSs) 1–5 and 6–10, respectively, and are connected by a hinge region. In this organization, the channel resembles a clamshell that encompasses a central hourglass-shaped pore with a narrow constriction ring in the middle of the membrane. This ring is lined by hydrophobic amino acid residues and is proposed to prevent leakage of ions in the “closed” state. SecE embraces the SecY clamshell at the hinge side in a V-shaped manner. The third subunit, Sec61*β* is peripherally associated with the SecYE complex. The pore-like opening in the center is obstructed by a plug-like domain also termed TMS 2a that resides at the periplasmic side of the constriction ring. Thereby, it closes the pore on the extracellular face of the membrane. In the clamshell organization of SecY, the two halves contact each other via TMS 2, TMS 7, and TMS 8. The opening between TMS2 and TMS7/8 is termed the lateral gate and localizes at the front of the SecY pore. When opened, it may provide an exit path for hydrophobic polypeptide segments to enter the membrane. The lateral gate also fulfills an important role in the channel opening mechanism during protein translocation [16]. It is believed that insertion of the signal sequence into the lateral gate region results in a widening of the central constriction and an opening of the channel. This in turn will destabilize the plug domain that once released from the extracellular funnel will vacate a central aqueous path for polar polypeptides to cross the membrane. Because of the high conservation of the core subunits of the translocon, the proposed mechanism of channel opening is likely conserved in all domains of life [8]. In this respect, it is remarkable that the structural work with the archaeal SecYE*β* complex has been instrumental to define a unifying mechanism of protein translocation despite the fact that the exact details of this process have not been resolved in archaea as so far no *in vitro* translocation system has been established.

### 2.2. Transport of Folded Proteins Across the Cytoplasmic Membrane

The Tat pathway mediates the transport of protein in their folded state. This in particular, but not only, concerns cofactor containing proteins that fold and assemble in the cytoplasm. Typically, the bacterial Tat-pathway consists of three integral membrane proteins, TatA, TatB, and TatC. In archaea and in most Gram-positive bacteria, the Tat complex consists of only two components, TatA and TatC, whereas the third component TatB is missing [11]. In current models, TatBC is involved in the initial recruitment of a substrate while TatA, probably in concert with TatC, forms the pore through which the folded protein is transported across the membrane [17]. In most bacteria and archaea, the number of Tat substrates is relatively small as compared to the number of substrates that are translocated by the Sec pathway. However, in halophilic archaea the Tat pathway is the predominant route for protein secretion [18]. This requirement for the Tat-pathway is thought to be an adaptation to the high-salt environment that may interfere with protein folding inside of the cell. However, the halophilic bacterium *Salinibacter ruber* mostly secretes proteins via the Sec route [19] suggesting that the requirement for Tat is not an adaptation to high salt per se. Another unique feature of the Tat pathway in haloarchaea is that translocation is driven by the sodium motive force whereas in many other microorganisms, the proton motive force is used as a driving force [20]. It should be noted that in the bacterium *Streptomyces coelicolor*, many of the proteins that are typically secreted by the Sec-pathway utilize the Tat pathway instead [21].

Proteins are routed to either the Sec or Tat pathway by an N-terminal signal peptide that upon secretion is removed by a signal peptidase. The basic tripartite organization of the signal peptides utilized by these two pathways is very similar. The Sec and Tat signal peptides have a three-domain structure: a positively charged amino-terminal n-domain, a central hydrophobic h-domain, and a polar c-domain which contains a cleavage site for the signal peptidase [22]. Apart from the presence of a pair of arginines in a SRRXFLK (X = any  amino  acid) motif in the N-region of Tat signal peptides [23], there is no sequence homology in the other regions. The signal peptides of the three domains of life are functionally interchangeable [24]. Remarkably, about 60% of the Tat signal sequences in *Escherichia coli* are able to route proteins to the Sec translocation machinery as well [23]. In this respect, unfolded proteins are rejected by the Tat pathway [25], although some other studies suggest that the Tat pathway can handle intrinsically unfolded proteins [26].

### 2.3. Transport Across the Outer Envelope

The most outer border of the archaeal cell is usually a layer of crystalline protein, that is, the surface (S-) layer. The S-layer contains pore-like openings that have suggested to allow free passage of nutrients and other small molecules [1]. However, little is known on how proteins cross this barrier during secretion. Protein secretion across the outer envelope, the outer membrane, has been studied in great detail in didermic bacteria. A total of seven different systems have been recognized in these organisms and the protein secretion processes associated with these systems are termed type I-VII secretion. Archaea share components of some of these systems, but since types III, V, VI, and VII secretion seem to be absent from archaeal genomes, these will not be further discussed here. 

Type I secretion involves an ATP-binding cassette (ABC) transporter that via a cytoplasmic membrane bound fusion (or adaptor) protein (MFP) associates with an outer membrane pore [27]. These systems secrete proteins directly from the cytoplasm to the exterior of the cell. ABC type transporters are relatively abundant in archaea but most are involved in substrate uptake [11]. It is not clear if type I secretion exists in archaea. However, no homologues have been identified of the membrane fusion proteins and porin proteins are absent because of the lack of an outer membrane. Proteomic studies in thermophilic crenarchaea show that a significant portion of the exoproteomes concerns proteins devoid of signal sequences. For instance, in the thermoacidophile *Sulfolobus solfataricus* secretion of a superoxide dismutase has been reported [28], but the gene encoding this protein does not specify a signal sequence and thus it remains unknown how this protein is released from the cells. Therefore, it remains to be established whether the presence of signal sequenceless proteins in the external medium is the result of a specific protein secretion process or cell lysis [29–31].

Type II secretion systems of didermic bacteria consist of 12 to 16 proteins that assemble into a secretion apparatus that spans both the cytoplasmic and outer membrane. The genes coding for the secretion system are often arranged into a large operon. With type II secretion, substrate proteins are first translocated to the periplasm by either the Sec- or Tat pathway [32, 33]. These proteins fold into their native state in the periplasm and may even assemble into multisubunit protein complexes. Next, these folded proteins are translocated across the OM through a large pore termed the secretin. The targeting of proteins to the secretin is poorly understood. For example, *Pseudomonas aeruginosa* secretes various proteins, such as a lipase, an elastase, and exotoxin A, via its type II secretion systems but these substrates share no common recognition motif and it is generally believed that the secretin recognizes structural folds rather than amino acid sequences [32]. Transport through the secretin is believed to involve a pseudopilus, a short filament that assembles from subunits at the cytoplasmic membrane. It has been proposed that the pseudopilus acts as a kind of piston to push substrates through the secretin across the outer membrane [32]. Although archaea do not possess an outer membrane, their flagella and pili assembly systems contain subunits reminiscent to proteins in the type II secretion systems of bacteria and will be discussed in more detail below.

Type IV secretion systems are involved in the transport of effector proteins and of DNA, but are considered to be primarily protein exporters that secrete DNA through its attachment to a secreted protein [34]. Very recently the structure of the type IV secretion channel was solved. This structure that contains 4 different subunits spans the entire periplasmic space and resides in the cytoplasmic and outer membrane [35]. Conjugative plasmids containing some subunits of type IV secretion systems have been identified in crenarchaea only [36–39]. In these homologs of the cytoplasmic ATPase VirB4, the polytopic membrane protein VirB6 and the coupling protein VirD4 were identified, but these are significantly different than their bacterial counterparts. No details are known about their involvement in conjugative transfer of DNA in archaea. In the euryarchaeote *Haloferax volcanii,* it was reported that bidirectional chromosomal DNA transfer occurred during conjugation, and large structures (2 *μ*m long and 0.1 *μ*m wide) bridging cells were postulated to mediate DNA transfer [40]. However, the system mediating this transfer has not been identified. 

Yet another well-studied system in bacteria is the assembly machinery of type IV pili that are involved in a multitude of functions such as surface adhesion, cell-cell contact, autoaggregation, twitching motility, and DNA uptake [41]. Type IV pilins contain the so-called class III signal peptides that prior to the pilus assembly reaction are processed by PilD, a processing peptidase that also methylates the N-terminal phenylalanine of the mature pilin [42]. Up to 15 proteins are involved in the correct assembly of the pilins into the pilus structure, but the driving force for its assembly is provided by the cytoplasmic ATPase PilB. This process is antagonized by the action of the ATPase PilT causing the disassembly of the pilus. Interestingly, the archaeal flagellum biogenesis apparatus resembles a simplified type IV assembly machinery and different archaeal surface structures have been identified which belong to the same class [43] (more details will be discussed in the section about archaeal surface structures). 

All type II/IV secretion and type IV pili assembly systems contain a cytosolic ATPase that functions as a motor to drive secretion or assembly. Because of the similarity, these ATPases likely function by similar mechanisms and are evolutionary related [44]. Secretion ATPases assemble into a hexameric ring. The structure of the secretion ATPase GspE2 of *A. fulgidus* shows that the N-terminal domain alternates between a standing and laying down position, and it has been suggested that this process is driven by ATP and needed to deliver a piston-like movement that would drive the movement (or assembly) of a pilus [45]. The relative shift of the N-terminal domain is 10 Å which fits to the required movement of 10.5 Å for pilus assembly [45]. The genomes of most archaea contain genes specifying several type II/IV secretion ATPases [45]. These are often arranged in an operon together with genes encoding pilin-like proteins and a membrane protein. Therefore, it appears that the archaeal assembly systems are of a lower complexity than their didermic bacterial counterparts, at least lacking the outer membrane protein components. In this respect, they are more similar to those observed in monodermic bacteria.

## 3. Signal Peptides and Secretomes

Three different classes of signal peptides which are processed by their own designated signal peptidase have been recognized [46]. Class I signal peptides are cleaved at the C-domain by type I signal peptidases. Proteins containing class I signal peptides are typically released as soluble proteins or are, if they contain a C-terminal transmembrane helix, C-terminally embedded in the membrane [47]. Class II signal peptides are exclusively found in lipoproteins. Characteristic of class II signal peptides is a conserved cysteine that is present at the cleavage site. After cleavage of the signal peptide, the cysteine forms the N-terminal residue of the mature protein where it serves as a lipid attachment site to anchor the protein to the membrane [48]. In bacteria, several steps are involved in processing of the class II signal peptide. First, a diacylglyceryl group is attached to the cysteine. This reaction is catalyzed by prolipoprotein diacylglyceryl transferase. After this modification the signal peptide is cleaved by the type II signal peptidase. The final step, that is, the attachment of a lipid, is then executed by an apolipoprotein N-acyltransferase. Peculiarly, none of the proteins involved in processing of class II signal peptides have been identified in archaea, despite the presence of functional class II signal peptides [49]. In archaea, Sec and Tat signal peptides can be found in both class I or class II signal peptides [46, 48]. Class III signal sequences are processed at the N-domain by a specific membrane-integrated peptidase that eliminates the positively charged amino acids, thus, leaving the H-domain of signal peptide attached to the protein. This processing event occurs at the inner face of the cytosolic membrane, and because of the removal of the positive charges the translocation block is removed allowing the subsequent translocation of the pilin subunit for downstream assembly. The latter involves the H-domain that functions as an assembly scaffold to support the formation of a pilus or pseudopilus on the outside of the cell [41, 42]. In archaea, the best example of a class III signal peptide bearing substrate is flagellin, the subunit of the archaeal flagellum that is used for motility. The class III signal peptides are processed by a specialized peptidase, that is, the preflagellin peptidase that utilizes the same catalytic mechanism as the bacterial prepilin peptidases [50, 51]. However, in archaea, class III signal peptides are not only confined to flagellins, pilins, and/or pseudopilins but are also found in a variety of other extracellular proteins such as substrate-binding proteins or proteases [52]. 

The signal peptide plays a decisive role in initiating the secretion process. In co-translational protein secretion, the protein synthesizing ribosome is brought to the transport machinery by a protein-RNA complex called Signal Recognition Particle (SRP). The SRP binds to the signal peptide of the protein being synthesized and to the ribosome. The ribosome-SRP complex interacts with a membrane-associated SRP receptor and upon entry of the signal peptide into the Sec translocon the SRP and SRP receptor are released [53]. In eukaryotes, the SRP contains six proteins together with a 300 nucleotide RNA molecule, whereas the bacterial version is much simpler as it consists of one protein, Ffh, and a 113 nucleotide RNA molecule. The archaeal SRP is similar to the eukaryote SRP albeit much smaller. It consists of two essential components; the SRP54 protein and a~300-nucleotide-long RNA molecule and the nonessential accessory protein SRP19 [54]. The archaeal SRP receptor is more similar to the bacterial SRP receptor FtsY than to the eukaryotic SRP receptor that consists of two subunits, SR*α* and SR*β* [55]. 

### 3.1. The Secretome

Current knowledge of protein secretion and the advancement of proteomics led researchers to define the secretome [56] which is the collection of proteins that is secreted by the cell. Essentially, these are the proteins that contain a signal peptide and that are actively transported across the cytoplasmic membrane, but proteomic studies have also identified sets of secreted proteins that do not contain an identifiable signal peptide but still can be regarded as secreted. In principle any program able to detect the presence of signal peptides can be used to create an *in silico* secretome. For example, PSORTb predicts the cellular localization of a protein and SignalP predicts the likelihood that a protein contains a signal peptide [57, 58]. By means of these prediction programs, various *in silico* secretomes of archaea have been drafted [30, 46, 59–61]. These vary from 1.2 up to 19% of the total proteome depending on the specific program, stringency of criteria, and the archaeal species analyzed. Of special interest are the programs PRED-SIGNAL and Flafind [52, 62]. PRED-SIGNAL has been designed exclusively for the prediction of archaeal signal peptides, while it also distinguishes between signal peptides and amino-terminal transmembrane helices. Analysis of 48 archaeal genomes by PRED-SIGNAL predicts that 5%–14% of the proteome specifies signal peptide-containing proteins, while no significant differences between crenarchaea and euryarchaea were found [62]. The program Flafind recognizes class III signal peptides, which in archaea are believed to be particularly important for the biogenesis of cell surface appendages. Flafind indicated the presence of 308 class III signal peptide-bearing proteins amongst 22 archaeal proteomes [52]. The majority of the Flafind positives are hypothetical proteins that are associated with pilus assembly systems. 

A critical issue is the experimental validation of the *in silico* secretomes. In the supernatant of the psychrophile *Methanococcoides burtonii* only 7 signal peptide-containing proteins have been identified [47]. In a later study, this number was increased to 16 proteins by applying a whole proteome analysis [63]. In *S. solfataricus, *attempts to cover the whole proteome resulted in the identification of 32 proteins exclusively present in the supernatant [31]. When an inventory was made of supernatant proteomes and cell surface subproteomes of three *Sulfolobus* species, a total of 64 proteins was reported [29]. In these *Sulfolobus* species, cell surface proteins dominated the supernatant proteome suggesting that actual secretion is a rare event and that the majority of the secreted proteins originate from cell surface released proteins. This notion was further strengthened by the observation that an extracellular *α*-amylase mostly resides at the cell surface [29]. Similar observations were made in the crenarchaeon *Aeropyrum pernix* in which 107 proteins were identified from both the cell surface and the supernatant [30]. The proteomic studies demonstrate that there are significant differences between predicted and experimental secretomes. For example, proteins devoid of an identifiable signal peptide are not predicted by the *in silico* methods but appear in large numbers extracellularly. An important source of proteins without signal peptides are those associated with extracellular membrane vesicles that appear to result from a specific secretion phenomenon (discussed below). It has been suggested that cytosolic proteins are secreted via yet unknown secretion systems [30], but this phenomenon appears general in proteomic studies in both bacteria and archaea and often concerns different proteins. Overall, these cytosolic proteins may be highly resistant against proteolysis and, therefore, show a long retention time in the external medium after cell lysis. None of the proteomic studies has achieved a full coverage of the *in silico* secretome. The latter is due to various limitations in the analysis. Often only one growth condition is used, and thus only a subset of proteins is expressed. Also, the methods are not optimized for the isolation of the extracellular cell surface associated proteins, and only those are observed that are released. By isolating the glycosylated cell surface proteins using lectin columns [29, 64], the set of identified extracellular proteins may be significantly expanded.

## 4. Membrane Vesicles as a Novel Secretion Vehicle

A rather unusual and poorly understood protein secretion mechanism is the release of proteins packaged into small membrane vesicles that emerge from the cell surface. Many didermic bacteria are known to release outer membrane vesicles from their surface [65], but this process also seems to occur in archaea where the membrane vesicles are coated with S-layer proteins. In a screen for viruses amongst the euryarchaeal order of *Thermococcales* it was discovered that most of the strains tested released small spherical vesicles [66]. These vesicles do not resemble viruses and often have genomic DNA associated to their surface [66]. Membrane vesicle release has been reported for many different archaea, such as the thermophilic euryarchaeon *Aciduliprofundum boonei* isolated from hydrothermal deep-sea vents [67], and various crenarchaeota, in particular *Sulfolobus * [68, 69]. With *S. islandicus* [70] and *S. tokodaii* [68] (Ellen et al, unpublished), the membrane vesicles appear to contain an antimicrobial protein(s) that inhibits the growth of related *Sulfolobus* species. The antimicrobial activity involves a proteinaceous component, but its identity has not yet been elucidated. Overall, it seems that in *S. tokodaii,* the antimicrobial protein(s) is specifically sorted to the membrane vesicles, but it is unknown if membrane vesicle formation is mechanistically linked to the secretion of the antimicrobial protein factors. Also *Ignicoccus* species are vigorous producers of membrane vesicles. These organisms lack a cell wall and instead contain an outer membrane-like structure. Electron microscopic investigations indicate that membrane vesicles are released from the cytoplasmic membrane and released in the spacious periplasmic space [2]. It has been suggested that these vesicles fuse with the outer membrane and that they are either part of a specific secretion system or involved in the biogenesis of the outer membrane. 

**Figure 2:**
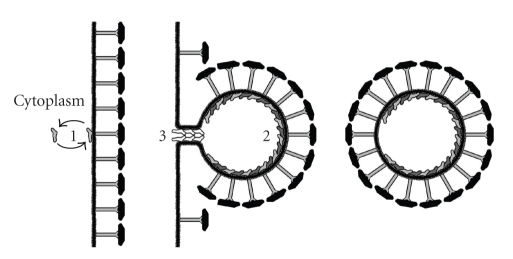
Model for vesicle budding in crenarchaea. Archaeal homologues of eukaryote ESCRT-III subunits are in equilibrium between a freely diffusible state in the cytoplasm and a membrane-bound state (1). If the equilibrium shifts towards the membrane associated state a heterocomplex (2) of different ESCRT-III subunits is formed leading to the creation of an outwardly growing bud that is covered by S-layer protein. Recruitment of the last group of ECRT-III subunits (3) creates the “neck” through which the bud is attached to the cytoplasmic membrane just before the membrane vesicle is pinched off and released into the medium.

To date, only for the *Sulfolobus* derived vesicles a proteomic analysis has been performed. The protein composition of these membrane vesicles is markedly different from that of the cytoplasmic membrane [68] suggesting that they may emerge from a specific release event. However, the vesicles do not seem to contain a specific cargo that would point to a specific role, except for the presence of archaeal homologues of the eukaryotic endosomal sorting complex required for transport-I (ESCRT) proteins [68]. This has led to the hypothesis that the membrane vesicles emerge from the cytoplasmic membrane through an outward budding event similar to the inward budding of vesicles in the endosomal compartment of eukaryotes (see Figure 2). The *Sulfolobus* vesicles vary in size from 50 to 200 nm and are surrounded by a S-layer, as verified by proteomic analysis and electron diffraction [70]. The presence of the S-layer coat indicates that the membrane vesicles are pushed through the cell envelope, which would be consistent with an assumed flexibility of the S-layer. The ESCRT-III proteins have also been implicated in cell division [71], and another possibility would be that the membrane vesicles are remnants of the cellular constriction and released during the cell division processes. Intriguingly, ESCRT-III proteins are not present in euryarchaea, although membrane vesicle formation has also been observed in these archaea.

The release of membrane vesicles appears a general feature observed in all three domains of life. In this respect, despite the presence of a cell wall, membrane vesicle release has also been reported for monodermic bacteria and fungi [72, 73]. In didermic bacteria, release of outer membrane vesicles is commonly observed feature and some indirect genetic evidence suggests that this is an essential process [74]. The protein composition of the outer membrane vesicles (or blebs) differs significantly from that of the outer membrane, suggesting that proteins are specifically sorted to the vesicles [75]. The exact function of membrane vesicle release has remained obscure as they have been implicated in a variety of processes. The membrane vesicles may function as a protein secretion system to provide a protected environment for the cargo. For instance, in *E. coli* *α*-haemolysin is secreted via a type I secretion system. However, the majority of the *α*-haemolysin remains tightly associated with outer membrane vesicles that also contain TolC, the outer membrane porin associated with the haemolysin type I secretion system. This suggests a link between the secretion of a membrane active toxin and membrane vesicle formation [76]. Membrane vesicle release may be a stress phenomenon providing a means to get rid of excess membrane material. In many cases, DNA seems to be associated with the membrane vesicles. For *Thermococcales*, it has been suggested that the associated DNA is not specifically packaged into the membrane vesicles but rather associates with the membrane vesicles after their release into the medium [66]. The DNA may originate from lysed cells, and because of the membrane association, it may become resistant to nuclease activity and, thus, show a greater persistence. Finally, membrane vesicle release may provide a means to secrete insoluble hydrophobic substances that partition into the lipid membrane. For example, many microorganisms produce quorum-sensing molecules with hydrophobic acyl chains of varying lengths. In *Pseudomonas aeruginosa* such quorum-sensing molecules are packaged into outer membrane vesicles [77]. The release of membrane vesicles could also serve to restore cellular imbalances caused by aggregates of denatured proteins as suggested for *E. coli* [78]. Future studies should reveal the exact function of the secreted membrane vesicles in archaea and provide clues on their mechanism of biogenesis.

## 5. Assembly of Archaeal Surface Structures

### 5.1. Archaeal Flagella: Structure and Function

Archaeal flagella have been studied at the genetic, structural, and functional level for several archaeal strains. Early observations of these pili-like filaments by electron microscopy led to the suggestion that they are functionally analogous of bacterial flagella performing similar tasks in swimming motility and biofilm formation. Cell motility by flagella has been demonstrated for the archaea *Halobacterium salinarum*, *M. voltae*, *S. acidocaldarius *and* S. solfataricus [79–83]*. In *H. salinarum*, the bidirectional rotation of the flagellum creates a motion to forward or reverse direction by instant switching of the flagellum rotation which appears to be similar to the rotation of bacterial flagellum [82]. Such a rotational motion has not yet been observed for other archaeal flagella. The flagella are also essential for surface attachment and colonization as demonstrated for *Pyroccocus furiosus* and *S. solfataricus* [84–86]. 

The subunit composition, structure, and assembly mechanism of the archaeal flagellum is very different from that of the bacterial flagellum [87, 88]. The archaeal flagellum has a right-handed helical subunit packaging with a diameter of approximate 10–14 nm which is much thinner than the bacterial flagellum [80, 89]. Only in few cases thicker filaments were found depending on the flagellins assembled [90]. The archaeal flagellum is not hollow and the inner space is most probably formed by coiled-coil interaction of the N-terminal hydrophobic domains of the flagellins similar to the assembled type IV pilus [91]. Moreover, recent studies suggest that the energy required for the rotation of the *H. salinarum* flagellum is directly gained from ATP hydrolysis and not from the proton motive force. Therefore, the mechanism of the *H. salinarum* flagellum rotation is fundamentally different from that of the bacterial system [92]. The archaeal flagellum is encoded by the *fla* operon, a single locus of 8–10 genes present in many Crenarchaeota and Euryarchaeota. The overall composition of the *fla*-operon shares homology with bacterial type-IV pili assembly, type II and type IV secretion systems [52, 80, 93–96]. Flagellins are the subunits of the flagellum and contain a class III signal peptide that is necessary for their membrane insertion and assembly into the flagellum. Processing involves the membrane peptidase FlaK (or PibD) [51, 97], and these enzymes are homologous to the bacterial PilD but do not catalyze the N-methylation of the newly formed N-terminus of the flagellin subunit. The H-domain likely folds into an extended hydrophobic *α*-helix that participates in coiled-coil interactions between subunits within the inner core of the flagellum. Reconstruction studies of the *H. salinarum* and *S. shibatae* flagella suggests that the H-domains constitute a central hydrophobic core similar to that of type-IV pili, but there is no direct evidence for a structural role of the H-domain [98, 99]. 

Archaeal flagella differ in the number of the structural subunits, the flagellins. The *fla* operon of *M. voltae* contains 4 structural flagellin genes: *flaA*, *flaB1*, *flaB2,* and *flaB3 *[100]. FlaB1 and FlaB2 are the major components of the flagellum and the deletion of their corresponding genes results in flagellum deficiency. FlaA is distributed throughout the flagellum as a minor component and deletion of *flaA* results in flagellated but less motile mutants [81]. FlaB3 is localized proximal to the cell surface forming a curved shape structure with similarity to the bacterial hook structure. Deletion of *flaB3* resulted in flagellated and motile mutants [101]. The similarity between this suggestive archaeal hook structure and the hook domain of bacterial flagella may indicate that a similar torque-driven motion is generated by the *M. voltae* flagellum. However, the mechanism of *M. voltae* motility is unknown and the role of the archaeal hook in rotation of the flagellum has not been demonstrated. In *H. salinarum*, five *fla* genes in two loci (*flaA1*, *flaA2* and *flaB1*, *flaB2, flaB3*) encode flagellum subunits [102–104]. The *flaA1* and *flaA2* genes encode the major components of the flagellum. The flagellum of *H. salinarum* does have a bi-directional rotation mechanism which drives the cells forward and backwards [82]. 

Possibly, the central core complex encoded by the *fla*-operon is only involved in assembly of the flagellum much akin that of bacterial type IV pilins, while another as yet unknown system functions as the rotating motor. The *Sulfolobales fla* operon contains only one structural flagellin gene, FlaB [80, 105]. In *P. furiosus*, FlaB1 is the main component of the flagellum, but the *fla* operon contains a second flagellin subunit (FlaB2) with unknown function [84]. FlaI is homologous to the bacterial type IV pili assembly and type II secretion ATPases, PilB and GspE, respectively. This further suggests a conserved mechanism for assembly of the archaeal flagellum and bacterial type IV pili assembly/type II secretion systems [89, 94–96]. ATPase activity was demonstrated for *S. solfataricus* and *S. acidocaldarius* FlaI proteins expressed and purified after overexpression in *E. coli* [94, 106]. So far, FlaI is the only identified ATPase component of the flagellum core complex and although its role in flagellation has been demonstrated with the deletion of the *flaI* gene, it remains unclear if FlaI is also involved in energizing the motility of the cell. FlaJ is the only known integral membrane component of the flagellar assembly system [79, 80, 101]. FlaJ proteins contain 9 transmembrane segments and two large cytoplasmic domains of about 25 and 15 kDa, respectively. These polar domains are thought to function as the interaction site for FlaI as shown for the membrane anchoring proteins of bacterial type II secretion systems. Structural analysis of the interacting domains of EpsE and EpsN, the assembly ATPase and the membrane protein of the toxin type II secretion system of the bacterium *Vibrio cholerae,* indicated that hydrophobic interactions and salt bridges are responsible for this interaction [107]. Alignment of archaeal FlaI/FlaJ with EpsE/EpsN suggests that this interaction might be conserved in the archaeal type IV pili assembly systems. The function of FlaJ in flagella assembly has not been examined. Although the flagellum of *S. solfataricus* is essential for motility on surfaces [80], a rotational motion and a hook-like structure in the flagellum filament remain to be demonstrated. Overall, the mechanism for twitching motility by means of the archaeal flagellum is poorly understood. 

The function of the other components of the archaeal flagellum assembly operon is unknown, however, in *H. salinarum,* it was recently demonstrated that the flagella accessory proteins FlaCE and FlaD interact via two newly identified proteins with three different proteins from the Che signaling cascade (CheY,CheD, and CheC2), providing the link between the flagellum and the sensory apparatus [108]. As Che proteins are lacking in crenarchaeotes also the FlaCEDs are absent in the flagella operon implying a different mechanism for how stimuli will be transduced into a change of motility direction.

### 5.2. Novel Archaeal Surface Structures

Archaea exhibit a wide variety of cell surface appendages with intriguing structures and biological functions. These appear to be highly specialized due to the specific adaptation of the microorganisms to their hostile habitats. The cannulae network of *Pyrodictium abyssi* is an example of such a structure [109, 110]. 

*P. abyssi* has been isolated from hydrothermal marine environments and its optimal growth temperatures range from 80 up to 100°C [111, 112]. The cannulae network seems crucial for cell survival as it is highly abundant in the cell colonies. Cannulae tubes have an outside diameter of 25 nm and they consist of at least three different, but homologous, glycoprotein subunits with identical N-termini but with different molecular masses (i.e., 20, 22, and 24 kDa). These proteins are highly resistant to denaturing conditions such as exposure to temperatures up to 140°C. From the three-dimensional reconstruction of the cannulae-cell connections, it appears that cannulae enter the periplasmic space but not the cytoplasm forming an intercellular connection of the periplasmic spaces between cells [109]. These connections are formed when cells divide whereupon the cells stay connected through the growing cannulae [111]. The function of the cannulae network is still unclear. It might act to anchor cells to each other or function as a means of communication, mediate nutrients exchange, or even transport of genetic material [87]. It is also not known which system(s) is (are) involved in the assembly of the cannulae network.

Another unusual archaeal cell surface appendage is the “hamus” [87, 113]. This structure represents a novel filamentous cell appendage of unexpectedly high complexity. Archaeal cells bearing these structures are found in macroscopically visible string-of-pearls-like arrangements which also entangle bacterial cells mainly *Thiothrix* (SM) or IMB1 proteobacterium (IM) that grow in cold (10°C) sulfidic springs [114]. The archaeal cells are coccoids of approximately 0.6 *μ*m in diameter with about 100 filamentous hami attached to each cell. Hami are 1 to 3 *μ*m in length and 7 to 8 nm in diameter and have a helical structure with three prickles (each 4 nm in diameter) emanating from the filament at periodic distances of 46 nm. The end of filament is formed by a tripartite, barbed grappling hamus-like hook. The hamus is composed mainly of a 120-kDa protein. However, the sequence of this protein is unknown. They are stable over a broad temperature (0 to 70°C) and pH range (pH 0.5 to 11.5) and mediate strong cellular adhesion to surfaces of different chemical compositions. It is proposed that the hami function in surface attachment and biofilm initiation, much like flagella and pili in bacterial biofilm formation, but in addition provides a strong means of anchoring.

A new pili type was recently isolated from *Ignicoccus hospitalis* which are 14 nm in width and up to 20 *μ*m in lenth and constitute up to 5% of cellular protein. They are composed mainly of protein Iho670, which has a class III signal peptide [115]. As *I. hospitalis* has an outer membrane, it would be expected that the pili assembly would be located in the outer membrane instead of the inner membrane as in all other known archaea. 

*S. solfataricus* expresses UV-induced pili at its cell surface [116]. This system is encoded by the *ups* operon and present in all S*ulfolobales* genomes [94]. This operon is strongly induced when *S. solfataricus* is exposed to UV light; subsequently the cells assemble pili at their surface and form large cellular aggregates. The Ups pili are much shorter than the wave-shaped flagella of *S. solfataricus *and are relatively thin with a diameter of 7 nm [80]. They show a right-handed helical symmetry similar to the flagellum. Mutants lacking the *upsE* gene that encodes a GspE-like ATPase are deficient in pili formation and cell aggregation. UpsE shares strong homology with FlaI and other assembly ATPases, and it likely energizes the assembly of the Ups pili. The *upsF* gene encodes the transmembrane protein of the assembly system and is it highly homologous to FlaJ. Another gene in the operon is *upsX*. UpsX shows no homology with any other protein and its function is unknown. The *ups* operon contains two genes that encode pilins, UpsA and UpsB. Both proteins contain a class III signal peptide and are processed by the general class III signal peptidase PibD. Overexpression of UpsA in *S. solfataricus* results in the formation of unusual long pili. Interestingly, the Ups pili are also essential for surface adhesion of *S. solfataricus* [86]. The Ups system and the flagellum can initiate the attachment of *S. solfataricus* to different surfaces and recent studies on *Sulfolobales* biofilm formation reveal the Ups system is essential for lateral biofilm formation (Koerdt and Albers, unpublished). 

Recent studies on the flagella and novel pili structures promoted an initiative to map archaeal pili-like biogenesis clusters through bioinformatics analysis of a large number of sequenced archaeal genomes [52]. The FlaFind program was developed to search for proteins containing class III signal sequences, which therefore encode putative structural surface proteins. This *in silico* analysis identified 388 putative class III signal sequence-containing proteins in 22 archaeal genomes, from which 102 proteins were annotated with a function: 44 flagellin subunits and 33 as substrate-binding proteins. Also extra cellular proteases and redox proteins were among this list. A total of 120 of these proteins were found connected to operons similar to bacterial type IV pilus assembly systems and type IV pilin signal peptidases. The FlaFind hits were analyzed for short and highly conserved motifs. Also eight additional SBP and 19 euryarchaeal proteins containing a QXSXEXXXL motif with unknown function were identified. In the DUF361 domain, the Q residue was at +1 from the cleavage site. Several of these proteins were identified in an operon together with a novel type IV signal peptidase called EppA from euryarchaeal *Methanococcus maripaudis*. Experiments showed that EppA specifically processes proteins belonging to the DUF361 group. The cleavage was tested by coexpressing a DUF361-containing protein with FlaK and EppA. It is probable that the DUF361 proteins are functionally and structurally different than the well-known flagellin and pilin proteins due to the requirement of a homologue but yet different type IV signal peptidase for the cleavage of their signal peptide. Recently, the structure of the *M. maripaudis* pilus has been resolved with cryo electron microscopy and it revealed a novel structure assembled from two subunit packaging [117]. A one-start helical symmetry filament and a ring structure of 4 subunits were combined in the same filament. 

Another intriguing archaeal type IV pilus assembly system is the bindosome assembly system (Bas) in *S. solfataricus* which is involved in assembly of sugar-binding proteins into the bindosome, a structure that is expected to be localized close to the cytoplasmic membrane or integrated within the S-layer [118]. The main evidence in support of the presence of this hypothesized structure is that the proposed structural components, the substrate-binding proteins (SBPs), contain class III signal peptide sequences, a feature typical of proteins which are well known to form oligomeric structures in both archaea and bacteria. The oligomerization of sugar-binding proteins was studied after isolation of the sugar-binding proteins from the membrane of *S. solfataricus* on size exclusion chromatography (Zolghadr et al., unpublished). Previous studies demonstrated that the precursors of the sugar-binding proteins are processed by PibD, the archaeal type IV signal peptidase [50, 97]. The sugar binding-protein oligomer is proposed to play a role in facilitating sugar uptake, a function that enables *S. solfataricus* to grow on a broad variety of substrates. 

The Bas system is unique and it has only been identified in *S. solfataricus*. The *bas* operon contains five genes that are organized into 2 smaller operons: the *basEF* genes encoding the main components of the assembly system which are homologues of FlaI/FlaJ of archaeal flagellum assembly system and UpsE/F from the Ups system of Sulfolobus [94]. A second set of genes encompasses *basABC* that encodes small pili-like proteins with class III signal peptides. BasABC is unique and has only been identified in *S. solfataricus*. Previous studies showed that they are constitutively expressed but the electron microscopic investigations did not reveal any pili structure assembled by BasABC. The uptake of glucose was strongly inhibited in a *basEF *deletion mutant and, concomitantly, growth on glucose was strongly impaired. However, the deletion of *basABC* only moderately affected the growth rate and sugar uptake. These results suggested that the Bas system is a novel assembly system involved in correct localization of sugar-binding proteins to the cell envelope, which have a pilin signal peptide. BasEF forms the core of the assembly machinery in the membrane while the BasABC assists the assembly of the binding proteins by an as yet unresolved mechanism.

## 6. Extracellular Polysaccharides

Bacteria secrete glycosylated proteins and exopolymer substances (EPSs) into the medium for the synthesis of extracellular structures and biofilm. EPS formation, not to be confused with protein glycosylation, is the assembly of long sugar polymers from diverse monosaccharides such as glucose, mannose, and fructose. The EPS is in most cases produced as a capsule surrounding the cell and thereby increasing the adhesion to surfaces or strengthening cell-cell contacts in cell aggregates which leads to biofilm formation [119–121]. Other roles of EPS within biofilms are mainly to provide stability for the structures of the biofilm and protection against different contaminants in media like heavy metals and toxic organic compounds. EPS production is in general increased when cells are exposed to contaminants. EPS and biofilm formation by archaea is a new research area. Using fluorescently conjugated lectins, it was demonstrated that surface attached *S. solfataricus* cells produced EPS containing a variety of different sugars (glucose, mannose, galactose, and N-acetylglucosamine) [86]. Interestingly, the extracellular network produced by PBL2025, a deletion strain appeared different to the wild-type strain *S. solfataricus* P2 strain. PBL2025 lacking a set of 50 genes, which are by BLAST-search analysis predicted to be involved in sugar metabolism/catabolism and transport of solutes across the cytoplasmic membrane. The disruption of these genes has led to the overproduction of EPS and an analysis of the expression pattern of these genes in P2 demonstrated that they are upregulated during surface attachment of the cells on mica [86], identifying the first genes involved in modulation of secreted polysaccharides. Most of the secreted archaeal proteins are glycosylated, a process that is described in detail by Eichler and Jarrell in this issue.

## 7. Conclusions and Outlook

Electron microscopic investigations of cultured and uncultivable archaea have revealed a remarkable variety of cell surface associated appendages. In recent years, the development of genetic systems for a number of model archaea now allows for experimental investigations on the assembly and function of these structures in at least some organisms. These studies now rapidly increase our understanding on how the archaeal cell surface is assembled. Various cell surface structures such as pili and flagella have been identified and their roles in cell-to-cell and cell-surface interactions start to be uncovered. Interestingly, also secreted vesicles have been identified in different archaeal species that contain a specific subset of proteins implied in an eukaryotic-like vesicle budding systems. This exemplifies the mosaic nature of archaea, which in many cases employ simplified eukaryotic-like mechanisms implying a similar evolutionary origin.
